# Artesunate Activates the Intrinsic Apoptosis of HCT116 Cells through the Suppression of Fatty Acid Synthesis and the NF-κB Pathway

**DOI:** 10.3390/molecules22081272

**Published:** 2017-08-08

**Authors:** Xiao Chen, Yin Kwan Wong, Teck Kwang Lim, Wei Hou Lim, Qingsong Lin, Jigang Wang, Zichun Hua

**Affiliations:** 1 The State Key Laboratory of Pharmaceutical Biotechnology, School of Life Sciences, Nanjing University, Nanjing 210023, China; pyb025@126.com; 2 Department of Biological Science, National University of Singapore, Singapore 117543, Singapore; e0146526@u.nus.edu (Y.K.W.); dbslimtk@nus.edu.sg (T.K.L.); whou327@gmail.com (W.H.L.); dbslinqs@nus.edu.sg (Q.L.); 3 Changzhou High-Tech Research Institute of Nanjing University, Institute of Biotechnology, Jiangsu Industrial Technology Research Institute and Jiangsu TargetPharma Laboratories Inc., Changzhou 213164, China

**Keywords:** artesunate, fatty acid biosynthesis, HCT116, mitochondrial apoptosis, NF-κB pathway, proteomic analysis

## Abstract

The artemisinin compounds, which are well-known for their potent therapeutic antimalarial activity, possess in vivo and in vitro antitumor effects. Although the anticancer effect of artemisinin compounds has been extensively reported, the precise mechanisms underlying its cytotoxicity remain under intensive study. In the present study, a high-throughput quantitative proteomics approach was applied to identify differentially expressed proteins of HCT116 colorectal cancer cell line with artesunate (ART) treatment. Through Ingenuity Pathway Analysis, we discovered that the top-ranked ART-regulated biological pathways are abrogation of fatty acid biosynthetic pathway and mitochondrial dysfunction. Subsequent assays showed that ART inhibits HCT116 cell proliferation through suppressing the fatty acid biosynthetic pathway and activating the mitochondrial apoptosis pathway. In addition, ART also regulates several proteins that are involved in NF-κB pathway, and our subsequent assays showed that ART suppresses the NF-κB pathway. These proteomic findings will contribute to improving our understanding of the underlying molecular mechanisms of ART for its therapeutic cytotoxic effect towards cancer cells.

## 1. Introduction

Colorectal cancer is one of the most common cancers worldwide with high mortality rates [1]. It was the most prevalent cancer in men and the second most in women between 2006 and 2010. Due to the high incidence rates of age-dependent colorectal cancer, several approaches have been determined to achieve positive anticancer effects, including surgery, chemotherapy, and radiotherapy. However, none of these therapies can produce the desired therapeutic effect when applied alone [2,3].

Recently, considerable attention has been paid to the development of new natural anti-cancer compounds to inhibit the multistep tumorigenesis with minimal adverse effects [4,5], such as resveratrol [6], curcumin [7,8], camptothecin [9] et al. Among these anticancer natural products, terpenes and their derivatives occupy an important position. Several studies show that pentacyclic triterpenes exert effects of antioxidant, antiproliferation, antimigration, and pro-apoptotic capacities in cancer cells [10,11,12], revealing their anticancer potentials of terpenes and their derivatives.

Artemisinin, a sesquiterpene lactone derived from an ancient Chinese herbal remedy, has been applied in chills and fever treatment for at least 2000 years [13]. Currently, artemisinin and its derivatives are regarded as the most potent anti-malarial drugs. Besides its antimalarial activity, increasing evidences suggest that artemisinin and its derivatives also exhibit anticancer activity in various cancer cell lines [14,15], such as colon cancer cells [16], breast cancer cells [17], oral cancer cells [18], and prostate cancer cells [19]. Depending on the cell line and experimental system, artemisinin affects a variety of processes in cancer cells, such as cell proliferation, apoptosis, and cellular hormone secretion.

Artesunate (ART), a derivative of artemisinin, apart from its antimalarial activity, also exerts potent cytotoxic effect against many human cancer cell lines, such as pancreatic cancer cells [20], neuroblastoma cells [21], breast cancer cells [22], and non-small cell lung cancer cells [23]. Several in vitro reports have demonstrated that ART induces a pleiotropic response in cancer cells, including inhibition of cell proliferation via cell cycle arrest [24], apoptosis [25], angiogenesis inhibition [26], disruption of cell invasiveness [27], and modulation of nuclear receptor responsiveness [28]. In addition, ART is also utilized in combination with other anticancer agents to reach a better therapeutic effect [29].

Although reports have showed that ART affects multiple processes in cancer cells, only very few studies have used a large-scale approach, such as proteomics [30,31], to explore ART’s mechanism of action for its anticancer effect [32]. This results in a lack of comprehensive understanding of its underlying anticancer mechanism [33,34]. In this study, we apply a quantitative proteomics approach to identify differentially expressed proteins between HCT116 cells with or without ART treatment, and elucidate the mechanism of action of ART’s anti-proliferation effect on HCT116 colorectal cancer cells with proteomic data analysis and in vitro validation.

## 2. Results

### 2.1. Artesunate Inhibits HCT116 Cell Proliferation in a Dose- and Time-Dependent Manner

**Figure 1:**
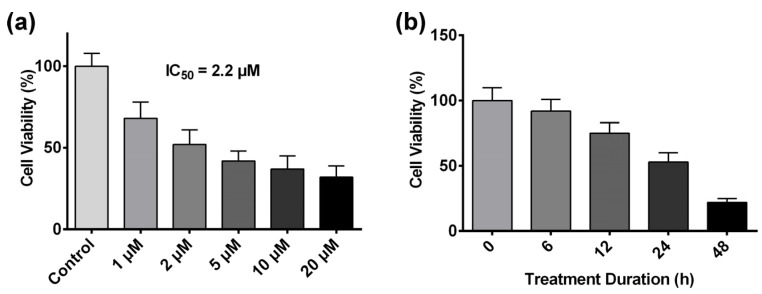
(**a**) HCT116 cell viability after treatment of different concentrations of artesunate (ART) for 24 h; (**b**) HCT116 cell viability after treatment with 2.2 μM ART for different span of time from 0 to 48 h.

Previous studies have demonstrated that colorectal cancer is the most susceptible cancer cell type to ART’s cytotoxic effect [35]. Hence, the HCT116 human colon carcinoma cell line was chosen as the tumor cell model to investigate ART’s mechanism of action in this study. The viability of HCT116 cells treated with ART was tested with crystal violet assay. It was shown that ART significantly inhibited HCT116 cell viability in a dose-dependent manner (Figure 1a), and the IC_50_ value (the concentration at which ART treatment inhibits a 50% growth of cell population) of ART on HCT116 cells was identified at 2.2 μM after 24 h of drug treatment. Based on this result, we adopted 2.2 μM as our experiment concentration in subsequent assays. HCT116 cells were also treated with 2.2 μM ART for different time-span (from 0 to 48 h), and ART inhibited HCT116 cell viability in a time-dependent manner (Figure 1b).

### 2.2. Using a Quantitative Proteomics Approach to Detect Protein Expression Alteration in ART-Treated HCT116 Cells

**Figure 2:**
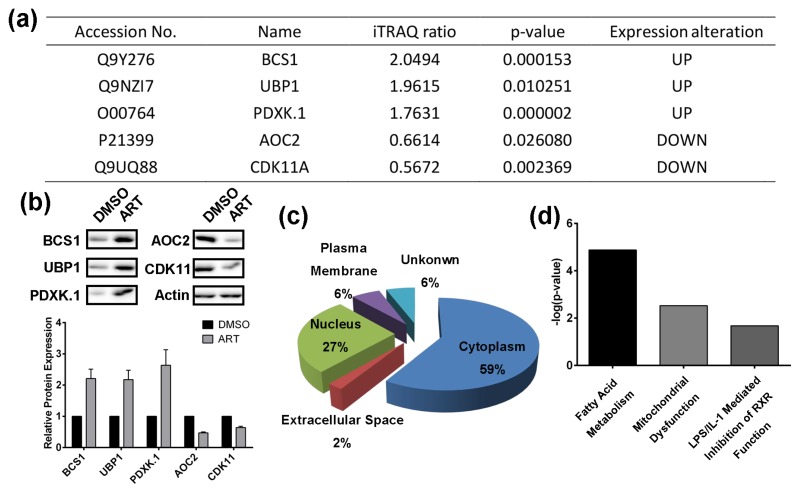
(**a**) Representative proteins modulated by ART in HCT116 cells; (**b**) Western-blotting validation of the selected ART-modulated proteins; (**c**) Ingenuity Pathway analysis of cellular localization of the ART-modulated proteins; (**d**) Top altered toxicity pathways regulated by ART in HCT116 cells.

In order to study the anti-cancer mechanism of ART, isobaric tags for relative and absolute quantification (iTRAQ) coupled with liquid chromatography (LC)-tandem mass spectrometry (MS/MS) was adopted to identify differentially expressed proteins between HCT116 cells treated with DMSO or ART for 24 h (Figure S1). Briefly, HCT116 cells were treated with ART or DMSO (control) for 24 h in parallel, followed by cell lysis and protein digestion. After iTRAQ labelling, the peptides of the two groups were pooled together and analyzed with LC-MS/MS to identify differentially expressed proteins. To ensure the specificity of the results, a strict cutoff threshold was employed: *p*-value < 0.05 and iTRAQ ratio for upregulated proteins > 1.3, while for downregulated proteins < 0.77. 123 proteins were identified to be the differentially expressed proteins from a total of 1540 quantified proteins identified. Among them, 61 proteins were upregulated by ART, while the expression of 62 proteins decreased after ART treatment. To further validate the identified proteins as the ART-modulated proteins, five representative proteins in our list, including mitochondrial chaperone BCS1 (BCS1), upstream-binding protein 1 (UBP1), pyridoxal kinase (PDXK.1), cytoplasmic aconitate hydratase (AOC2), and cyclin-dependent kinase 11A (CDK11A), were subjected to immunoblotting with their respective antibodies (Figure 2a,b). All the five proteins are involved in a variety of cellular processes responsible for cell viability and proliferation [36,37,38,39]. Results unequivocally confirmed the regulating effect of ART on the proteins.

We then conducted the Ingenuity Pathway analysis (IPA) to predict ART-modulated cellular pathways. The regulated proteins are distributed broadly in different parts of the cells, and especially in the cytoplasm (Figure 2c). 59% of the identified proteins are localized in the cytoplasm, suggesting that ART might perform its anticancer actions by affecting cytosolic protein activity. IPA analysis also showed that ART might initiate anticancer effects over two critical biological pathways, namely the fatty acid metabolism pathway and the mitochondrial dysfunction pathway (Figure 2d).

### 2.3. Artesunate Suppresses the Fatty Acid Biosynthetic Pathway to Inhibit Cancer Cell Proliferation

**Figure 3:**
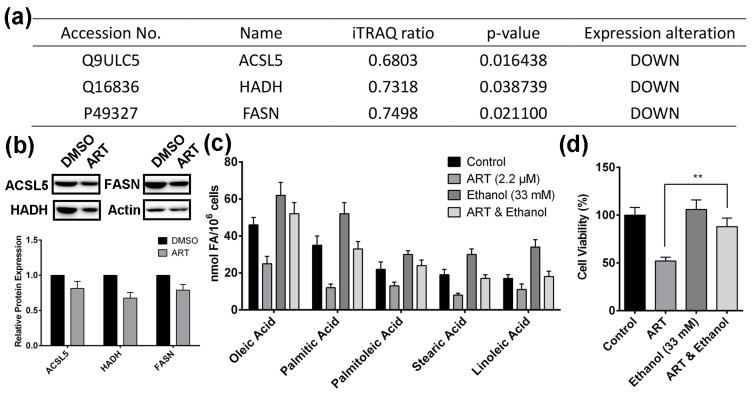
(**a**) Fatty acid biosynthesis-related proteins down-regulated by ART in HCT116 cells; (**b**) Western-blotting validation of fatty acid biosynthesis related proteins; (**c**) Fatty acid content alteration in HCT116 cells with ART treatment; (**d**) The effect of ethanol on ART-treated HCT116 cell viability; (** *p* < 0.01).

In our study, fatty acid metabolism is interpreted as the most perturbed pathway regulated by ART. Therefore, we hypothesized that ART inhibits HCT116 cell proliferation through suppressing fatty acid biosynthetic pathway. Figure 3a lists ART-regulated proteins that mediate fatty acid biosynthetic pathway, including Acyl-CoA synthetase 5 (ACSL5), hydroxyacyl-coenzyme A dehydrogenase (HADH), and fatty acid synthase (FASN). ART in HCT116 cells down-regulated all the three proteins. Results from western blotting confirmed that ART actually down-regulated the three proteins in HCT116 cells (Figure 3b). ACSL5 is an essential enzyme catalyzing the formation of acyl-CoA [40]; HADH plays a pivotal role in fatty acid metabolism and the decreasing expression of HADH leads to the disruption of β-oxidation pathway [41]; FASN catalyzes the key step in producing crucial biological molecule, palmitate [42]. As oleic acid (OA), palmitic acid (PA), palmitoleic acid (PO), stearic acid (SA), and linoleic acid (LA) are the five most abundant fatty acids in HCT116 cell lines [43], we did GC/MS analysis to determine fatty acid content alterations in HCT116 cells with ART treatment (Figure 3c). The results showed that ART significantly decreased the content of these fatty acids in HCT116 cells. In summary, ART inhibits the fatty acid biosynthetic pathway in HCT116 cells.

Next, we sought to determine whether ART-induced fatty acid inhibition affects HCT116 cell proliferation. Previous reports showed that ethanol up-regulated the expression of sterol regulatory element-binding protein (SREBP) [44], which is the activator of the complete program of fatty acid synthesis [45]. Ethanol treatment alone significantly increased the content of fatty acid in HCT116 cells, and ethanol completely reversed the ART-induced decrease of fatty acid content (Figure 3c). In addition, ethanol alone did not affect HCT116 cell viability, but rescued cells from ART’s cytotoxic effect (Figure 3d), suggesting that the inhibitory effect of ART on fatty acid synthesis contributes to ART’s anti-proliferation activity.

### 2.4. Artesunate Treatment Results in ROS Production and Mitochondrial Apoptosis Pathway Activation in HCT116 Cells

**Figure 4:**
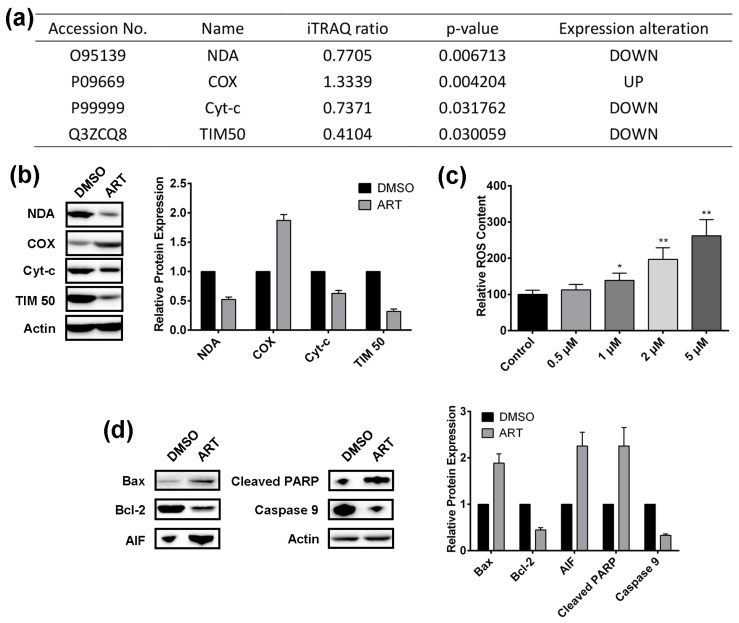
(**a**) ART modulated proteins involved in mitochondrial dysfunction in HCT116 cells; (**b**) Western-blotting validation of proteins involved in mitochondrial dysfunction; (**c**) The effect of different concentrations of ART on reactive oxygen species (ROS) content in HCT116 cells; (**d**) The effect of ART on the expression of key signaling molecules of the mitochondrial death pathway; (* *p* < 0.05; ** *p* < 0.01).

Mitochondrial dysfunction has been ranked as the top two cytotoxic actions induced by ART (Figure 2d). NADH dehydrogenase (NDA), Cytochrome c oxidase (COX), Cytochrome c (Cyt-c), and mitochondrial inner membrane translocase (TIM50) in our ART-modulated protein list are involved in mitochondrial function (Figure 4a). The modulating effect of ART on the proteins was also validated by western blotting (Figure 4b). ART up-regulated NDA, Cyt-c, and TIM50, while decreasing the expression of COX in HCT116 cells. NDA is reported to reduce the production of reactive oxygen species (ROS) from mitochondria [46], Cyt-c is released from mitochondria in a ROS-dependent fashion and can operate as a ROS scavenger [47], and TIM50 is recognized as important for regulation of mitochondrial integrity and cell death [48], and can regulate ROS [49]. Hence, we hypothesized that ART may induce ROS production to inhibit HCT116 cells.

DCFH-DA was employed to detect the ROS level, and the results showed that ART significantly increased the ROS level in HCT116 cells in a dose-dependent manner (Figure 4c). Next, as TIM50 regulates mitochondrial integrity and cell death, we sought to examine whether ART treatment modulates the expression of key signaling molecules of the mitochondrial death pathway. Results from western blotting showed that ART significantly up-regulated Bax, AIF, and cleaved-PARP expression, while decreasing the expression of Bcl-2 and caspase 9 (Figure 4d). Reports showed that Bax functions as an apoptotic activator [50]; AIF, named apoptosis inducing factor, is involved in initiating a caspase-independent pathway of apoptosis [51]; and cleaved PARP and caspase 9 cleavage are the markers for mitochondrial-mediated apoptosis [52]. Bcl-2 is specifically considered an important anti-apoptotic protein [53]. Therefore, we conclude that ART activates the mitochondrial apoptosis pathway in HCT116 cells.

### 2.5. Artesunate Treatment Inhibits the Nuclear Factor (NF)-κB Pathway

**Figure 5:**
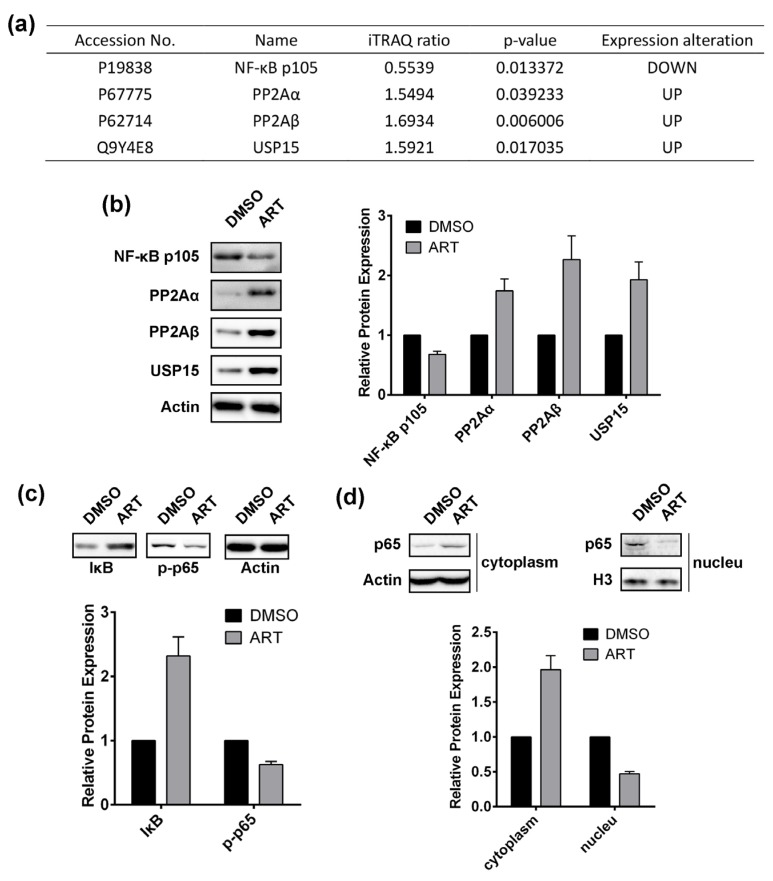
(**a**) ART-modulated proteins involved in NF-κB pathway in HCT116 cells; (**b**) Western-blotting validation of proteins involved in NF-κB pathway; (**c**) Effect of ART on the expression of IκB and phosphorylated NF-κB p65 subunit; (**d**) Abundance alteration of NF-κB p65 subunit in cytoplasm and nucleus of HCT116 cells with or without ART treatment.

Apart from fatty acid biosynthesis inhibition and mitochondrial dysfunction, we also discovered that ART could regulate the expression of several proteins involved in the NF-κB pathway, including NF-κB p105 subunit, serine/threonine-protein phosphatase 2A catalytic subunit alpha isoform (PP2Aα), serine/threonine-protein phosphatase 2A catalytic subunit beta isoform (PP2Aβ), and ubiquitin carboxyl-terminal hydrolase 15 (USP15) (Figure 5a). ART down-regulated NF-κB p105 expression, while up-regulating the expression of PP2aα, PP2Aβ, and USP15, which were validated by western blotting (Figure 5b). Reports showed that PP2A inhibits the NF-κB pathway [54], and that the PP2A inhibitor okadaic acid leads to slow activation of IKK and consequently NF-κB [55]. In addition, USP15 was also proved to abrogate the pro-survival NF-κB activity [56]. Therefore, we inferred that ART might inhibit the NF-κB pathway in HCT116 cells.

In order to corroborate the effect of ART on the NF-κB pathway, we applied western blotting to determine the expression of IκB and phosphorylated NF-κB p65 subunit (p-p65) in HCT116 cells with or without ART treatment (Figure 5c). Results showed that ART significantly increased IκB expression but down regulated p-p65, suggesting that ART inhibits the NF-κB pathway in HCT116 cells. We also extracted the proteins in the cytoplasm and the nucleus of HCT116 cells, respectively, and discovered that in the cytoplasm ART increased NF-κB p65 subunit expression, whereas it was decreased in the nucleus (Figure 5d), demonstrating that ART inhibits the NF-κB p65 subunit translocating to the nucleus, which indicated that ART indeed suppresses the NF-κB pathway. As NF-κB is a nuclear transcription factor that modulates expression of a large amount of genes responsible for the regulation of apoptosis [57], ART might inhibit HCT116 cell proliferation through suppressing the NF-κB pathway.

## 3. Discussion

Artemisinin and its analogs are widely recognized as effective antimalarial drugs. Recently, artemisinin and its derivatives extended their role from a well-established antimalarial drug to a promising anticancer agent. Extensive research has demonstrated their potent antitumor activity in the nano- to micromolar range in sensitive cancer cells, such as leukemia and colorectal cancer [35,58]. In order to adapt artemisinin and its derivatives into a more effective and safer cancer therapy, substantial studies have been conducted to elucidate the molecular mechanisms of action for the cytotoxic effect of these compounds [59]. Artesunate (ART), a derivative of artemisinin, besides its antimalarial activity, also shows potent cytotoxic effect against a variety of human cancer cell types. However, all the postulated mechanisms of action for ART have not been broadly accepted. Hence, to unravel the biological pathways that are perturbed by ART, it is crucial to implement effective strategies that would provide a detailed picture of all the differentially expressed proteins after drug treatment using large-scale proteomic profiling.

**Figure 6:**
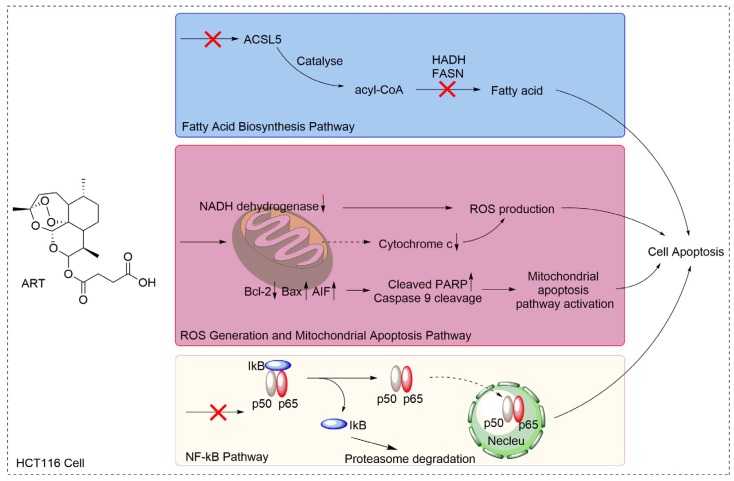
Proposed mechanism for the proliferation inhibitory effects of ART in HCT116 cells.

Here, we first evaluated the effect of ART on HCT116 cell proliferation and discovered that ART inhibited HCT116 cell proliferation in a concentration- and time-dependent manner. The inhibitory effect of artemisinin on human colon cancer cells has been broadly reported [60,61]. ART, a derivative of artemisinin, also exerts an inhibitory effect on a variety of cancer cell lines, such as pancreatic cancer cells, neuroblastoma cells, breast cancer cells, lung cancer cells, and others. Although reports showed the anticancer activities of ART on human colon cancer cells, which are in line with our findings, very few of them applied a large-scale proteomic profiling approach to investigate the mechanism, resulting in a lack of a comprehensive understanding of ART’s MOA on colon cancer cells. For instance, Li et al., reported that ART inhibited human colon carcinoma cell growth through the Wnt/β-catenin pathway without an investigation of ART’s effect on other cellular processes and signaling pathways [62]. In the present study, we used the advantages of a high-throughput, quantitative proteomics approach to identify differentially expressed proteins in ART-treated HCT116 cells, and screened all the cellular processes and signal pathways that might be affected by ART with proteomic analysis. From the results, we discovered that ART affected various cellular processes and pathways, including fatty acid biosynthesis, ROS generation, the mitochondrial apoptosis pathway, and the NF-κB pathway involved in cell proliferation (Figure 6).

In cancer pathogenesis, endogenous fatty acid biosynthesis is substantially enhanced to provide an essential building block for phospholipid membrane, conferring cancer cells growth advantages [63]. Pharmacological inhibition of fatty acid synthesis by several compounds, such as Orlistat, has been considered as a potentially effective cancer therapy [64], indicating that fatty acid synthesis is an effective target for anticancer agents. In the study, three fatty acid synthesis-related proteins were identified to be down regulated by ART. ACSL5 catalyzes the formation of acyl-CoA, which is required for cell proliferation and provides a substantial fatty acid source for membrane phospholipids [65]. In addition, ACSL5 also facilitates the transportation of exogenous fatty acids into cancer cells by cooperating with fatty acid transporters [65]. HADH is a key protein in fatty acid metabolism and the inhibition of HADH leads to fatty acid metabolism suppression. Moreover, HADH has been found to be extensively up-regulated in colorectal cancer cells [66], implying that fatty acid metabolism in HCT116 cells is excessively activated and fatty acid synthesis is important for colorectal cell proliferation. Therefore, the inhibition of fatty acid synthesis is a potential target in colorectal cancer therapy. FASN is the key lipogenic enzyme that catalyzes the formation of an important molecule, palmitate, and high expression of FASN has been observed in carcinogenesis [67]. Moreover, down-regulation of FASN and inhibition of FASN activity offer a promising therapeutic anticancer effect by inducing tumor cell-cycle arrest and cancer cell death [68,69]. Taken together, we hypothesized that fatty acid synthesis in HCT116 cells is an important process for cell proliferation, and ART suppresses fatty acid synthesis in HCT116 cells to inhibit cell proliferation. In our subsequent assays, we discovered that fatty acid content in ART-treated HCT116 cells significantly decreased, proving that ART suppresses fatty acid synthesis in HCT116 cells. Moreover, when we used ethanol to activate the fatty acid synthesis in ART-treated HCT116 cells, the content of fatty acid recovered and the cell viability was rescued from ART cytotoxicity to some extent, demonstrating that fatty acid is essential for cell proliferation and that ART suppresses HCT116 cell proliferation partially through the inhibition of fatty acid synthesis.

Mitochondrial dysfunction is one of the top toxicity pathways induced by ART from our IPA results. Several studies suggested that mitochondria is involved in ART-induced cell death in other cell lines [70,71,72]. Therefore, we hypothesized that ART induces mitochondrial dysfunction to inhibit cell proliferation in HCT116 cells. In supporting this hypothesis, our western blotting results showed that ART up-regulated expression of Bax, AIF, and cleaved PARP, and down-regulated Bcl-2 and caspase 9, suggesting that ART activates the mitochondrial apoptosis pathway. In addition, the proteins involved in mitochondrial function (NDA, COX, Cyt-c, and TIM50) in our list are also related to ROS production. As ROS generation is an important therapeutic target to kill cancer cells, many reports have applied agents that promote ROS production to treat cancer [73,74,75]. What’s more, several studies reported that ART treatment-induced ROS-mediated oxidative stress, leading to subsequent apoptotic cell death [20,22,71]. For instance, Efferth et al., found that ART induces ROS generation to induce T leukemia cells’ apoptosis [25]. Therefore, we concluded that ART promotes ROS production to induce HCT116 cells death. The results showed that ART increased the ROS level in HCT116 cells in a concentration-dependent manner, which confirmed our hypothesis.

NF-κB is a transcriptional factor that regulates the expression of many genes involved in various cellular pathways, such as cytokines, growth factors, anti-apoptotic molecules, and microRNAs. Constitutive activation of NF-κB has been implicated in the pathogenesis of various cancers, such as ovarian cancer, hepatocellular carcinoma, breast cancer, lung cancer, and prostate cancer [76]. Studies showed that blockade of the NF-κB pathway led to the inhibition of cancer cell proliferation, invasion, and metastasis [77,78]. Furthermore, a large amount of reports have employed anti-NF-κB agents to treat cancer cells [79,80]. Of interest, reports have showed that ART suppressed tumor growth and progression through inhibiting the NF-κB pathway in many cell lines, including prostate cancer cells, human cervical carcinoma cells [81], and human erythroleukemic cells [82]. Notably, according to our iTRAQ results, the NF-κB pathway was shown to be inhibited upon ART treatment, suggesting that ART affects the NF-κB pathway to suppress HCT116 cell proliferation. The conclusion was validated by western blotting.

In our previously published paper [32], we demonstrated that ART is activated by heme to exert its antiproliferation activity [83,84], and it displays very low cytotoxicity on normal colon cells (CCD841 colon cell) due to the low heme level in normal cells. This suggests that ART can inhibit colon cancer cell proliferation selectively. To conclude, our study reinforces current understanding of the anticancer activity of ART and elucidates the mechanism of ART’s cytotoxicity on HCT116 cancer cells, providing evidence that ART can be further developed as a potential anti-cancer agent in the future.

## 4. Materials and Methods

### 4.1. Materials

HCT116 human colon carcinoma cells were obtained from the American Type Culture Collection (CCL-247). Artesunate is purchased from Sigma Aldrich (A3731, St. Louis, MO, USA). Antibodies for western blotting are purchased from Cell Signaling Technology (Danvers, MA, USA) and Abcam (Cambridge, UK): BCS1 (Abcam; ab102808), UBP1 (Abcam; ab30965), PDXK.1 (Abcam; ab119051), AOC2 (Abcam; ab83734), CDK11 (CST; 5524), Actin (CST; 3700), ACSL5 (Abcam; ab57210), HADH (Abcam; ab54477), FASN (CST; 3180), NDA (Abcam; ab81212), COX (Abcam; ab110267), Cyt-c (CST; 11940), TIM50 (Abcam; ab23938), Bax (CST; 5023), Bcl-2 (CST; 15071), AIF (Abcam; ab32516), Cleaved PARP (CST; 5625), Caspase 9 (CST; 9502), NF-κB p105 (CST; 12540), PP2Aα (CST; 2041), PP2Aβ (CST; 4953), USP15 (Abcam; ab56900), IκB (CST; 9247), p-p65 (CST; 3033), p65 (CST; 8242).

### 4.2. Crystal Violet Assay

HCT116 cells were cultured in 96-well plate for 24 h before exposure to varying concentrations of ART or ethanol. Medium was removed after incubation of ART and ethanol for different time spans. The cells were washed twice with phosphate-buffered saline (PBS), followed by crystal violet staining for 15 min. The cells were washed again and the plate was air dried. 1% SDS was used to solubilize the cells for 30 min and the absorbance was measured at 550 nm with a microplate reader.

### 4.3. Sample Preparation for iTRAQ Proteomic Approach

HCT116 cells were cultured and treated with vehicle (dimethyl sulfoxide DMSO) or 15 µM ART as reported previously for 24 h. After treatment, the cells were harvested and lysed with lysis buffer containing 0.5 M triethylammonium bicarbonate (TEAB) and 1% sodium dodecyl sulfate (SDS). Subsequently, the cell lysates were centrifuged at 14,500 rpm at room temperature for 1 h. The supernatants were collected as samples for iTRAQ labelling and stored at −80 °C until use.

### 4.4. iTRAQ Labelling

iTRAQ labelling of each sample was carried out according to the manufacturer’s protocol (Applied Biosystems, Foster City, CA, USA). Briefly, 100 µg of proteins from the respective cell lysates were incubated with tris-(2-carboxyethyl) phosphine (TCEP) and methyl methane-thiosulfonate (MMTS). After cysteine blocking, the samples were diluted 20 times and trypsinized at 37 °C for 16 h. The digested peptides were then incubated with respective iTRAQ reagent at room temperature for 2 h to be labeled. After incubation, all iTRAQ-labelled samples were pooled together into a single fresh tube. Strong cation exchange (SCX) chromatography was subsequently performed to remove any interfering substances. The eluate obtained from SCX was further desalted by using Sep-Pak C18 cartridges, followed by vacuum drying and reconstitution with 5 mM KH_2_PO_4_, 5% acetonitrile for 1D LC-MS/MS analysis.

### 4.5. 1D LC-MS/MS Analysis

Eksigent NanoLC-Ultra system coupled with cHiPLC-Nanoflex system was applied to separate peptides labelled with different iTRAQ reagents. Peptides were separated by a gradient formed by mobile phase A (2% ACN, 0.1% FA) and mobile phase B (98% ACN, 2% H20, and 0.05% FA) from 12–40% of mobile phase B in 90 min, at a flow rate of 300 nL/min. The MS analysis was conducted on a TripleTOF 5600 analyzer (AB SCIEX, Foster City, CA, USA). The MS spectra were collected across the mass range of 350–1250 *m*/*z*, using 250 ms accumulation time per spectrum. For each mass-spectrometry spectrum, a maximum of 20 precursors with a charge state between 2 and 4 were chosen for fragmentation. Also, the signals were accumulated for 100 ms per spectrum and dynamic exclusion time was set at 15 s. MS/MS spectra were measured in high sensitivity mode.

### 4.6. Peptide and Protein Identification, Data Analysis

ProteinPilot Software (4.5, AB SCIEX, Foster City, CA, USA) was applied to quantify and identify peptides. A randomized database generated by the Proteomics System Performance Evaluation Pipeline (PSPEP) was used to estimate the false discovery rate (FDR).

In this work, two biological replicates of control- and ART-treated samples were analyzed. Student’s *t* test was conducted and the *p*-values of each protein based on the iTRAQ ratio indicates the significance of differentially expressed protein. Only proteins with *p*-value < 0.05 (significantly different) were selected for further analysis. Subsequently, the significant cutoff thresholds used to determine up-regulated proteins and down-regulated proteins were 1.3 and 0.77, respectively. Therefore, identified proteins with an average iTRAQ ratio larger than 1.3 were considered as up-regulated proteins, while proteins that possess an average iTRAQ ratio smaller than 0.77 were considered as down-regulated proteins.

### 4.7. Western Blotting Assay

Proteins were extracted from cell lysate. 10% SDS-PAGE was applied to separate the proteins according to their molecule weight. After SDS-PAGE, proteins were transferred to a nitrocellulose membrane (Millipore, Billerica, MA, USA). Then the membrane was blocked with 5% milk, and followed by incubation with respective primary antibody and secondary antibody. ECL detection reagent (Thermo Scientific, Rockford, IL, USA) was employed to detect the proteins.

### 4.8. GC/MS Analysis for Fatty Acid Content

Samples were prepared according to published studies [85,86]. Cell lysate was dried in nitrogen atmosphere, and fatty acid within the lysate was transesterificated. Then, the fatty acid methyl esters were analyzed with GC/MS. The average content of fatty acids was calculated by multiplying the number of double bonds in fatty acids by the percentage of those compounds and dividing by 100.

### 4.9. ROS Content Analysis

HCT116 cells were treated with ART for 12 h and subsequently stained with DCFH-DA. Flow cytometry was applied to quantify the fluorescence signals [87].

### 4.10. Statistical Analysis

GraphPad prism (5.0, GraphPad Software, La Jolla, CA, USA) was used for statistical analysis. Data was summarized as mean ± SEM. One way ANOVA was used to determine the significant differences between groups. Results were considered to be significant for *p*-values of <0.05.

## 5. Conclusions

In conclusion, based on the cell-based study, we have first shown the cytotoxicity of ART on HCT116 cells. Next, in an effort to elucidate the molecular mechanisms of ART, the present study offers comprehensive proteomic profile of untreated and ART-treated HCT116 cancer samples by using an iTRAQ technique coupled with LC-MS/MS. In our study, we have characterized the anticancer effect of ART by exploring the functional categorization of differentially expressed proteins upon ART treatment. First, ART achieves its therapeutic anticancer effect by partially regulating the fatty acid biosynthetic pathway. In addition, ART evidently augments the production of ROS and activates the mitochondrial apoptosis pathway. Lastly, inhibition of aberrant NF-κB signaling upon ART treatment has been postulated from the definite biological functions of the respective altered candidates. Therefore, we proposed that several cellular perturbations occur in response to ART cytotoxicity, including the dysregulation of fatty acid biogenesis, ROS production, and mitochondrial apoptosis pathway activation, as well as modulation of the NF-κB pro-survival pathway. This study reinforces the current understanding of the anticancer activity of ART and could provide valuable insights to identifying potential molecular targets from those perturbed pathways, thus contributing to the development of ART as an effective antitumor agent for human cancer therapy.

## Supplementary Materials

The following are available online. Figure S1: General workflow of iTRAQ coupled with LC-MS/MS, Table S1: up-regulated proteins list, Table S2: down-regulated proteins list.
