# Supplementary material for: Artesunate Activates the Intrinsic Apoptosis of HCT116 Cells through the Suppression of Fatty Acid Synthesis and the NF-κB Pathway
Source: Molecules. 2017 Aug 8;22(8):1272. doi: 10.3390/molecules22081272 (PMC6152404; doi:10.3390/molecules22081272)
Supplement: Supplementary file 1 [file molecules-22-01272-s001.pdf]

Supplementary Material - Artesunate activates the intrinsic apoptosis of HCT116 cells through the suppression of fatty acid synthesis and the NF-κB pathway

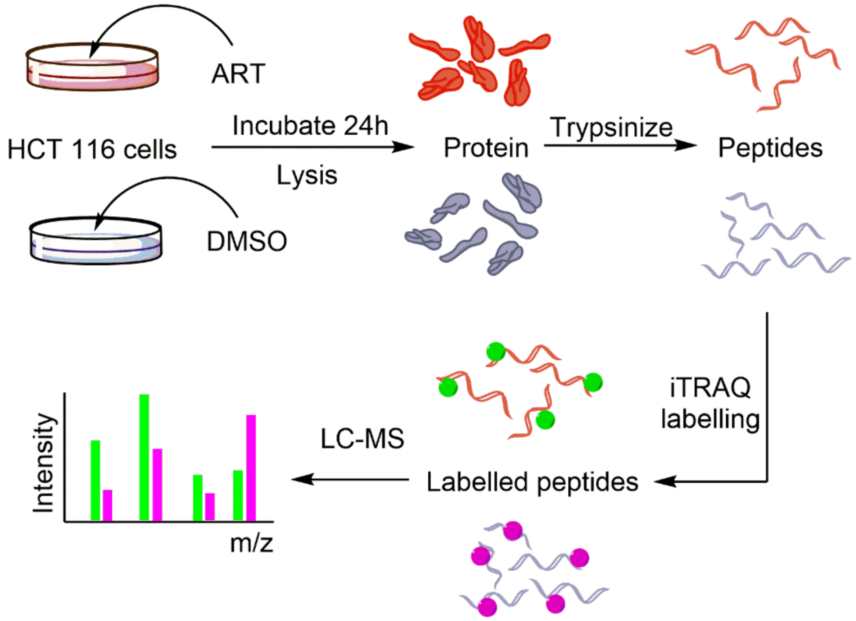

Figure S1 General workflow of iTRAQ coupled with LC-MS/MS

Table S1 up-regulated proteins list

Accession No.	Name	iTRAQ ratio	p-value
Q9NZN4	EH domain-containing protein 2	8.0099	0.031923
P06132	Uroporphyrinogen decarboxylase	5.8747	0.016095
P61026	Ras-related protein Rab-10	3.439	0.005855
Q9H078	Caseinolytic peptidase B protein homolog	2.7617	0.002846
Q16134	Electron transfer flavoprotein-ubiquinone oxidoreductase, mitochondrial	2.9526	0.030664
P49590	Probable histidine--tRNA ligase, mitochondrial	2.3891	0.002177
Q8N2F6	Armadillo repeat-containing protein 10	2.2024	0.000134
Q9Y276	Mitochondrial chaperone BCS1	2.0494	0.000153
Q14376	UDP-glucose 4-epimerase	2.0395	0.009034
O96019	Actin-like protein 6A	2.0094	0.007761
Q7Z7F7	39S ribosomal protein L55, mitochondrial	1.92	0.000000
Q96AT9	Ribulose-phosphate 3-epimerase	2.055	0.024096
A8MT69	Centromere protein X	1.955	0.007304
Q9NZI7	Upstream-binding protein 1	1.9615	0.010251
P61916	Epididymal secretory protein E1	1.84	0.000746
O00764	Pyridoxal kinase	1.7631	0.000002
Q68E01	Integrator complex subunit 3	1.7461	0.000000
P33240	Cleavage stimulation factor subunit 2	1.7262	0.013726
P62714	Serine/threonine-protein phosphatase 2A catalytic subunit beta isoform	1.6934	0.006006

Q5T3I0	G patch domain-containing protein 4	1.7312	0.032666
Q6P2E9	Enhancer of mRNA-decapping protein 4	1.6454	0.005915
O95400	CD2 antigen cytoplasmic tail-binding protein 2	1.6068	0.000281
Q9GZS3	WD repeat-containing protein 61	1.6132	0.018229
O95801	Tetratricopeptide repeat protein 4	1.5976	0.019642
Q9Y4E8	Ubiquitin carboxyl-terminal hydrolase 15	1.5921	0.017035
O75607	Nucleoplasmin-3	1.5703	0.005650
Q9ULC4	Malignant T-cell-amplified sequence 1	1.5265	0.002768
Q9Y5K5	Ubiquitin carboxyl-terminal hydrolase isozyme L5	1.5531	0.028656
Q12830	Nucleosome-remodeling factor subunit BPTF	1.5603	0.048384
P67775	Serine/threonine-protein phosphatase 2A catalytic subunit alpha isoform	1.5494	0.039233
O00217	NADH dehydrogenase [ubiquinone] iron-sulfur protein 8, mitochondrial	1.5025	0.000408
O43709	Uncharacterized methyltransferase WBSCR22	1.4841	0.011058
P18858	DNA ligase 1	1.4794	0.008162
P43490	Nicotinamide phosphoribosyltransferase	1.4622	0.000115
P04899	Guanine nucleotide-binding protein G(i) subunit alpha-2	1.4635	0.004454
Q96P70	Importin-9	1.4478	0.003815
Q9Y450	HBS1-like protein	1.4335	0.000006
Q96BM9	ADP-ribosylation factor-like protein 8A	1.4289	0.000006
Q9Y2A7	Nck-associated protein 1	1.4608	0.048551
Q9P0J0	NADH dehydrogenase [ubiquinone] 1 alpha subcomplex subunit 13	1.4147	0.001202
Q9NQ29	Putative RNA-binding protein Luc7-like 1	1.4168	0.002457
P11021	78 kDa glucose-regulated protein	1.4064	0.000004
P14854	Cytochrome c oxidase subunit 6B1	1.4449	0.047485
P11310	Medium-chain specific acyl-CoA dehydrogenase, mitochondrial	1.4338	0.035329
P30043	Flavin reductase (NADPH)	1.4039	0.003508
Q2NL82	Pre-rRNA-processing protein TSR1 homolog	1.3871	0.010745
P50552	Vasodilator-stimulated phosphoprotein	1.3727	0.000013
O43795	Unconventional myosin-Ib	1.3755	0.002744
Q9H0S4	Probable ATP-dependent RNA helicase DDX47	1.3934	0.034110
P36871	Phosphoglucomutase-1	1.3642	0.000000
P46926	Glucosamine-6-phosphate isomerase 1	1.3657	0.004824
O15269	Serine palmitoyltransferase 1	1.3818	0.038490
Q9NQ88	Fructose-2,6-bisphosphatase TIGAR	1.3613	0.007543
Q5VYK3	Proteasome-associated protein ECM29 homolog	1.3519	0.000153
P61960	Ubiquitin-fold modifier 1	1.3437	0.000002
O15173	Membrane-associated progesterone receptor component 2	1.3429	0.000932
Q96T88	E3 ubiquitin-protein ligase UHRF1	1.3357	0.000418
Q96I99	Succinyl-CoA ligase [GDP-forming] subunit beta, mitochondrial	1.3365	0.002639
P62249	40S ribosomal protein S16	1.3293	0.000006

P09669	Cytochrome c oxidase subunit 6C	1.3339	0.004204
O15145	Actin-related protein 2/3 complex subunit 3	1.3316	0.002179

Table S2 down-regulated proteins list

Accession No.	Name	iTRAQ ratio	p-value
P51648	Fatty aldehyde dehydrogenase	0.7774	0.027189
Q8IWA0	WD repeat-containing protein 75	0.769	0.001687
O95139	NADH dehydrogenase [ubiquinone] 1 beta subcomplex subunit 6	0.7705	0.006713
O14981	TATA-binding protein-associated factor 172	0.7768	0.043901
P05386	60S acidic ribosomal protein P1	0.7733	0.026063
Q9NPJ3	Acyl-coenzyme A thioesterase 13	0.7512	0.000160
Q9UBI1	COMM domain-containing protein 3	0.7509	0.000084
Q15102	Platelet-activating factor acetylhydrolase IB subunit gamma	0.7508	0.000159
P38606	V-type proton ATPase catalytic subunit A	0.7535	0.018759
Q96ST2	Protein IWS1 homolog	0.7552	0.026231
P49189	4-trimethylaminobutyraldehyde dehydrogenase	0.7572	0.041505
Q8TEX9	Importin-4	0.7508	0.026374
O60610	Protein diaphanous homolog 1	0.7502	0.024978
P49327	Fatty acid synthase	0.7498	0.021100
Q6ZRP7	Sulfhydryl oxidase 2	0.7354	0.000089
Q16204	Coiled-coil domain-containing protein 6	0.7347	0.002975
Q9NY33	Dipeptidyl peptidase 3	0.734	0.003457
P30837	Aldehyde dehydrogenase X, mitochondrial	0.7382	0.034906
P99999	Cytochrome c	0.7371	0.031762
Q16836	Hydroxyacyl-coenzyme A dehydrogenase, mitochondrial	0.7318	0.038739
O75616	GTPase Era, mitochondrial	0.7262	0.028356
P52655	Transcription initiation factor IIA subunit 1	0.7184	0.006370
Q9Y520	Protein PRRC2C	0.717	0.006595
Q96GA7	Serine dehydratase-like	0.714	0.010163
Q9H0U4	Ras-related protein Rab-1B	0.7057	0.019173
Q92688	Acidic leucine-rich nuclear phosphoprotein 32 family member B	0.6911	0.000237
Q8NF37	Lysophosphatidylcholine acyltransferase 1	0.7038	0.021787
Q9Y5Y2	Cytosolic Fe-S cluster assembly factor NUBP2	0.6955	0.005161
Q8IY37	Probable ATP-dependent RNA helicase DHX37	0.6903	0.001066
Q96JB5	CDK5 regulatory subunit-associated protein 3	0.7059	0.049627
O14776	Transcription elongation regulator 1	0.6974	0.025789
Q8TEM1	Nuclear pore membrane glycoprotein 210	0.6819	0.002978
O95340	Bifunctional 3'-phosphoadenosine 5'-phosphosulfate synthase 2	0.6747	0.000536
Q9ULC5	Long-chain-fatty-acid--CoA ligase 5	0.6803	0.016438
Q9Y6H1	Coiled-coil-helix-coiled-coil-helix domain-containing protein 2, mitochondrial	0.6784	0.022056

Q8IX18	Probable ATP-dependent RNA helicase DHX40	0.6584	0.000307
Q96TA1	Niban-like protein 1	0.6732	0.034846
P21399	Cytoplasmic aconitate hydratase	0.6614	0.026080
O43719	HIV Tat-specific factor 1	0.6465	0.020424
O60684	Importin subunit alpha-7	0.6352	0.010637
P19525	Interferon-induced, double-stranded RNA-activated protein kinase	0.6415	0.027415
Q9UPN3	Microtubule-actin cross-linking factor 1, isoforms 1/2/3/5	0.6505	0.048222
P30626	Sorcin	0.6154	0.000122
Q15021	Condensin complex subunit 1	0.6089	0.000002
Q9NRP0	Oligosaccharyltransferase complex subunit OSTC	0.5903	0.000084
Q14657	L antigen family member 3	0.5976	0.024880
Q15269	Periodic tryptophan protein 2 homolog	0.5975	0.030609
Q9UQ88	Cyclin-dependent kinase 11A	0.5672	0.002369
Q6L8Q7	2',5'-phosphodiesterase 12	0.5641	0.015498
Q9BUI4	DNA-directed RNA polymerase III subunit RPC3	0.5729	0.033112
P19838	Nuclear factor NF-kappa-B p105 subunit	0.5539	0.013372
Q9UET6	Putative ribosomal RNA methyltransferase 1	0.553	0.031663
Q8N9T8	Protein KRI1 homolog	0.4955	0.000170
Q9BVI4	Nucleolar complex protein 4 homolog	0.4787	0.000754
Q8TED0	U3 small nucleolar RNA-associated protein 15 homolog	0.4813	0.004463
P26196	Probable ATP-dependent RNA helicase DDX6	0.4832	0.009561
Q9H2P9	Diphthine synthase	0.4322	0.007755
Q9NRY5	Protein FAM114A2	0.4207	0.008607
Q3ZCQ8	Mitochondrial import inner membrane translocase subunit TIM50	0.4104	0.030059
Q8N183	Mimitin, mitochondrial	0.367	0.013408
P82675	28S ribosomal protein S5, mitochondrial	0.3353	0.003648
Q9HA64	Ketosamine-3-kinase	0.1756	0.000305
